# An open chat with…Simon Rayner

**DOI:** 10.1002/2211-5463.13543

**Published:** 2023-01-17

**Authors:** Ioannis Tsagakis, Simon Rayner

**Affiliations:** ^1^ FEBS Open Bio Editorial Office Cambridge UK; ^2^ Medical Genetics Oslo Universitetssykehus Norway; ^3^ Medical Genetics, Faculty of Medicine University of Oslo Norway

## Abstract

Simon Rayner joined the *FEBS Open Bio* Editorial Board in March 2022. Currently, he is Professor of Bioinformatics at Oslo University Hospital and the University of Oslo in Norway. He received a PhD in computational solid‐state physics from the University of East Anglia, in 1991, and served as a Lecturer at the Dept of Physics in the University of Texas before working as a Research Fellow at the McDermott Centre for Human Growth and Development, University of Texas Southwestern Medical Centre at Dallas (UTSW). He went on to become Assistant Professor at the Depts of Biochemistry & Internal Medicine, UTSW before co‐founding BioAutomation Inc. where he developed DNA & RNA synthesis technology and was involved in the Human Genome Project. Simon is a CAS scholar and has also been a Professor of Bioinformatics at the Chinese Agricultural University in Beijing. Here, he tells us about his multi‐disciplinary career transitions, including experience working on three continents, and reflects on implementing open science and fair data.

## Graphical abstract

Simon Rayner joined the *FEBS Open Bio* Editorial Board in March 2022. Currently, he is Professor of Bioinformatics at Oslo University Hospital and the University of Oslo in Norway. Here, he tells us about his multi‐disciplinary career transitions, including experience working on three continents, and reflects on implementing open science and fair data.

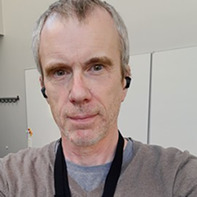

## What was it that first drew you to the non‐coding RNA field?

What I was interested in as a physicist was how systems respond to external influence. What I like about microRNAs (miRNAs) is that they're very imprecise in the way they regulate their targets, since they only partially bind to their target, meaning it's very hard to predict how or what a miRNA will regulate. So that means it puts a lot of noise into the system and I'm interested in how system noise is necessary for providing system stability.

I originally got involved because I was working on infectious disease and I was not only looking at how a particular strain of a virus was more virulent but also how evolution was effectively allowing the virus to probe and find niches where it could be more effective.

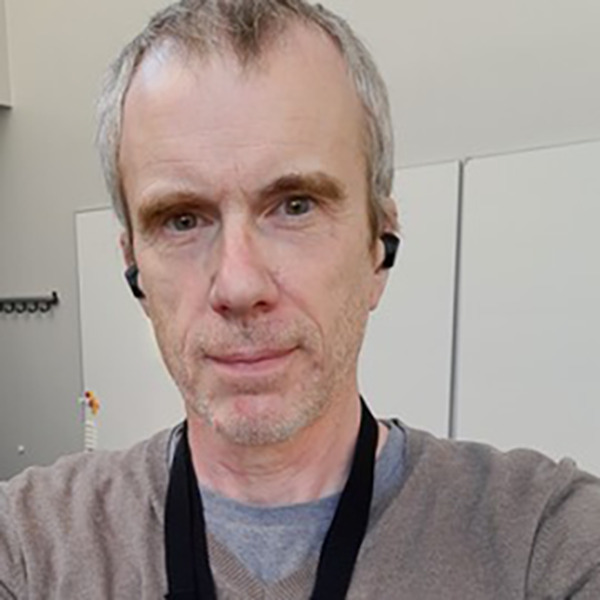

## What would you say were the biggest challenges you faced when you were first settling into your PhD?

I think the biggest one was I'd been a PhD student for 3 months and my advisor announced that he was moving to the United States. It was actually an interesting experience but then I had to strip the lab down into boxes, ship it to the United States, reassemble everything, and we had to do the translation from 240 volts to 110 volts. I had to go and rewire all the equipment and then set it all up again. So it took 18 months to actually get to the point where I could take measurements to start doing my PhD. While that was a challenge, it was also a really good experience, i.e., actually shifting a lab to a new country and then trying to set up. And I had a very good advisor who was a good mentor, so I think it was a good tradeoff.

## What inspired you to move into industry after starting a career in academia?

I think this comes back to not really having a clear career plan. So I've actually been back and forth three times between industry and academia. Because I was really trying to find something that interested me and it was only when I discovered infectious diseases that I realised this was what I really wanted to work on. I originally worked just doing software development in industry, and then I got offered a lecturer position at the university, and so I went back. Then I ended up getting involved in the Human Genome Project where I developed technology that we used to form a company, and so I went back to industry. Once the company was stable I fancied doing something else, so that's why I ended up in China, to just try something different. That was where I discovered infectious diseases. And then when I ended up leaving China, I used industry as a stepping stone again, until I got this current position as well [Professor of Bioinformatics at Oslo University Hospital and the University of Oslo in Norway].

## Why did you switch field from physics to biology?

That was actually because my sister was doing a PhD in molecular biology and I started reading about what she was doing to try and understand it. Then I became interested in that and I ended up moving into that field. Then a position came up working on the Human Genome Project, and it was originally developing robotics so that's how I ended up switching over. I enjoyed doing it, and then I ended up switching to bioinformatics.

## What are your impressions of working in academia versus industry?

I think it's changed a lot over the years, and now there's less difference between industry and academia. Nowadays, it seems there's pressure to produce both in industry and academia, in one, there's this pressure to publish, and in the other there's this pressure to develop the product. Whereas I think maybe when I was a student, it was more relaxed and you could actually do research and test the hypothesis. If you didn't publish something for a year or two, then as long as you were showing that you were working your way forward in your research then that was okay. But I think now the metric is how much have you published, where have you published, how much spending have you got. So now, I think this distinction is less clear.

## What would you say are the main differences in terms of research culture between the three continents (Europe, Asia and North America) you have worked on?

I worked both in Beijing and Wuhan and there was such a big difference in the culture. So even within China, moving from the north to the south was a big change. But certainly within China there's much more of a hierarchical structure, so much so that even I couldn't go and talk to the boss. Instead, I would have to talk to the next person up, then they will talk to next person, and they will talk to the person higher up. So you have to respect hierarchy, whereas in Norway, it is very different. I used to work in Norway and Denmark, both of which are very different in culture as well, and the situation there is also very different. It's a much flatter environment.

## What advice would you offer to anyone considering studying/working in labs in China?

When I was there, there were very few PhD programmes for foreigners, but people could come across for 3 months or so. I was the only foreigner in my institute and there were no foreigners around the city, or at least there were very few. So I never had any firsthand experience of what the PhD programmes were like when I lived there. But I think they now have good PhD programmes, there are very good labs and they have these joint programmes between American universities and Chinese institutes. So I think it's definitely something I would encourage people to consider, not just from the point of view of having the opportunity to work in good research labs, but just also the experience of being in a completely different culture; it's just a great experience.

## What do you like to do outside of the lab?

In Norway, it's always outdoor stuff since there are nice hills. People go skiing and cycling in the summer, so it's just a very good environment for being outdoors.

## Why did you decide to join the *FEBS Open Bio* Editorial Board?

Somebody contacted me and asked me if I was willing to do it. When I was in China, we'd taken this journal, *Virologica Sinica*, which was a Chinese language‐only journal, and we translated it to an English language journal. We actually also got an SCI entry as well, and it has an impact factor of 6.947 now. It was an interesting experience trying to make that transition and we realised that there were a lot of good manuscripts that would be rejected from journals, either based on language issues or because the editors decided they weren't interesting. For example, manuscripts talking about an infectious disease outbreak in a very local region in southeast Asia. At the time, we were doing some good research and we were aiming for publication. So we ended up publishing some very nice research and I got the sense that there was the same flexibility in *FEBS Open Bio*, the same way that we wanted to encourage research at *Virologica Sinica*. For *FEBS Open Bio*, the question when considering submissions is: “Is the research good?” So I just got that sense that, yes, it's probably worth trying to contribute to this journal. So that was my motivation.

## What are your views on open science and sharing data?

Obviously, I think open access is a great thing. I think that what tends to be overlooked is not the idea of open data but actually making the data available. It's not just about being “here's an Excel spreadsheet”. In an article we just published, we were trying to come up with a solution that is of help in actually sharing data in a fair‐like way (FAIR: data which meet the principles of findability, accessibility, interoperability, and reusability). The Norwegian Research Council, for example, and ERC state that people must indicate how they will implement fair data in their grant. However, the guidelines themselves are not always very clear. There's no clear way about how to show that your data are fair.

Even if you've published your article open access, making your data open as well is a big challenge. In addition, making your software available is equally challenging as well. That's one of the things that we're trying to work on as well, i.e. trying to provide software solutions to make data fair‐like for publication, but also for sharing amongst collaborators. This will help to get away from this idea of using Excel spreadsheets, and instead having a software platform where you can specify a set of standards that you can use for sharing your data amongst collaborators. In that way people could upload their data as part of the publication and then say these are the standards that we used in the data we are sharing.

For example, I think fairsharing.org has 1600 different standards, but actually it is not always straightforward or clear how you actually use those standards in your everyday work, and I think that's what's missing. I don't really think it's the responsibility of the journals to do that, because you'd then end up with different journals having different sets of standards or different platforms to implement those standards. What would be better is if there could be software that different journals could use open source, that would allow an author to provide the data, the standards and the platform they used.

